# Towards the fully automated monitoring of ecological communities

**DOI:** 10.1111/ele.14123

**Published:** 2022-10-20

**Authors:** Marc Besson, Jamie Alison, Kim Bjerge, Thomas E. Gorochowski, Toke T. Høye, Tommaso Jucker, Hjalte M. R. Mann, Christopher F. Clements

**Affiliations:** ^1^ School of Biological Sciences University of Bristol Bristol UK; ^2^ Sorbonne Université CNRS UMR Biologie des Organismes Marins, BIOM Banyuls‐sur‐Mer France; ^3^ Department of Ecoscience Aarhus University Aarhus Denmark; ^4^ UK Centre for Ecology & Hydrology Bangor UK; ^5^ Department of Electrical and Computer Engineering Aarhus University Aarhus Denmark; ^6^ BrisEngBio, School of Chemistry University of Bristol Cantock's Close Bristol BS8 1TS UK; ^7^ Arctic Research Centre Aarhus University Aarhus Denmark

**Keywords:** community ecology, computer vision, deep learning, high‐resolution monitoring, remote sensing

## Abstract

High‐resolution monitoring is fundamental to understand ecosystems dynamics in an era of global change and biodiversity declines. While real‐time and automated monitoring of abiotic components has been possible for some time, monitoring biotic components—for example, individual behaviours and traits, and species abundance and distribution—is far more challenging. Recent technological advancements offer potential solutions to achieve this through: (i) increasingly affordable high‐throughput recording hardware, which can collect rich multidimensional data, and (ii) increasingly accessible artificial intelligence approaches, which can extract ecological knowledge from large datasets. However, automating the monitoring of facets of ecological communities via such technologies has primarily been achieved at low spatiotemporal resolutions within limited steps of the monitoring workflow. Here, we review existing technologies for data recording and processing that enable automated monitoring of ecological communities. We then present novel frameworks that combine such technologies, forming fully automated pipelines to detect, track, classify and count multiple species, and record behavioural and morphological traits, at resolutions which have previously been impossible to achieve. Based on these rapidly developing technologies, we illustrate a solution to one of the greatest challenges in ecology: the ability to rapidly generate high‐resolution, multidimensional and standardised data across complex ecologies.

## Graphical abstract

Monitoring living organisms with high‐resolution and multidimensional is a complex and labour‐intensive task, yet it is fundamental to understand and predict the dynamics of ecological communities in an era of global change and biodiversity declines. Here, we review existing technologies for automated data recording and processing, and we present novel frameworks that combine these technologies into automated monitoring pipelines that detect, track, classify and count multiple species, and even record behavioural and morphological traits at resolutions which have previously been impossible to achieve. We illustrate a solution to one of the greatest challenges in ecology and conservation: the ability to rapidly generate high resolution, multidimensional and critically, standardised data across complex ecologies.

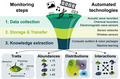

## INTRODUCTION

Ecosystems are increasingly exposed to stressors, leading to unprecedented rates of biodiversity decline globally (Capdevila et al., 2022; Díaz et al., 2019). Our ability to reliably forecast ecosystems dynamics is limited by our capacity to understand what governs their composition, dynamics, function and structure (Dietze et al., 2018; Petchey et al., 2015). To drive predictive ecology forward and design appropriate conservation strategies, we therefore need access to long‐term, high‐resolution and standardised information about ecosystems’ abiotic and biotic components (Farley et al., 2018; Mccord et al., 2021). Indeed, short time‐series and low‐resolution monitoring of a limited number of biological and ecological metrics can be detrimental to our understanding of ecosystems dynamics (White, 2019; White & Hastings, 2020; Wickham & Riitters, 2019) and are thus not recommended (Sparrow et al., 2020). In contrast, long‐term, high‐resolution and multidimensional data—from environmental parameters to individual morphological and behavioural traits, and up to species abundances, distributions and interaction—are key to holistically understand the mechanisms driving ecosystems dynamics (Naeem et al., 2016). The fine‐scale patterns present in these multidimensional data are particularly useful to predict potential population collapses and manage ecosystems accordingly (Cerini et al., 2022; Dietze et al., 2018). However, acquiring such data has traditionally involved cost‐prohibitive, labour‐intensive and often invasive survey methods that have consequently limited historical ecological observations both spatially and temporally (Kays et al., 2015; Pimm et al., 2015).

Recent technological advances in sensing technologies and their increasing accessibility have considerably improved our data collection capacity and are fundamentally changing how we sample ecological data (Allan et al., 2018). Using networked sensor arrays, environmental abiotic characteristics (e.g. humidity, light, pressure, temperature, pH) can already be monitored automatically, in real‐time, and over large spatiotemporal scales (Pansch & Hiebenthal, 2019; Urrutia‐Cordero et al., 2021). However, ecologists are also typically interested in complex biotic metrics such as the behaviours, locations and traits of individuals, as well as species abundances, distributions and interactions, which ultimately define ecological communities. Technologies such as acoustic sensors and camera traps can rapidly, remotely, non‐invasively and automatically collect high‐resolution sounds and images, thus replacing, augmenting and surpassing human sampling abilities (Cordier et al., 2018; Darras et al., 2019; Marcot et al., 2019; Wearn & Glover‐Kapfer, 2019; Welbourne et al., 2015). Nevertheless, processing such data into meaningful ecological measurements remains a challenging task to automate and a critical operational bottleneck (Keitt & Abelson, 2021). Traditional approaches have required significant human effort to examine features and patterns in sounds and images that correlate with ecological reality (Pimm et al., 2015). Such manual procedures do not scale efficiently with ever‐growing volumes of raw data produced by modern sensing technologies and are mostly inappropriate for large‐scale monitoring of complex ecosystems (Kindsvater et al., 2018; Peters et al., 2014).

To face this challenge, ecology increasingly relies on state‐of‐the‐art computational methodologies that automate data processing and knowledge extraction from ecological records (Farley et al., 2018). Over the last decade, artificial intelligence has revolutionised the way we use computers to identify features and patterns in ecological datasets automatically, accurately and reliably (Christin et al., 2019). Using computer audition, computer vision and machine learning algorithms, ecologists can today automate complex tasks covering the detection, identification, counting and measurement of individuals from images and audio recordings (Brodrick et al., 2019; Lürig, 2022; Mcloughlin et al., 2019; Peters et al., 2014). Deep learning, a branch of machine learning based on multilayer artificial neural networks, has been particularly successful at performing these tasks (Christin et al., 2019; Scholl et al., 2021; Tuia et al., 2022). While these approaches are becoming increasingly popular in ecology, their use often requires expertise from multiple disciplines (e.g. ecology, computer science, and electronic engineering), such that their potential is generally not realised. Indeed, these technologies have primarily been used: (i) on single species systems (e.g. to track and quantify multiple traits of a single individual or a single species population; Panadeiro et al., 2021; Walter & Couzin, 2021); (ii) on multiple species but at suboptimal resolutions (e.g. on camera trap images with low frame rates or short temporal coverage [Høye et al., 2021; Weinstein, 2018], or on a limited number of image features [Norouzzadeh et al., 2018]); or (iii) asynchronously (e.g. by processing data offline rather that in real‐time; Jarić et al., 2020). A more powerful approach would be to combine these data recording and processing technologies into accessible pipelines that could automatically and continuously monitor multiple species, in real‐time, with high‐resolution and multidimensional for long time periods (Christin et al., 2019).

Here, we begin by reviewing these technologies before exploring how they can be incorporated into pipelines that can generate high‐throughput multidimensional data for accurate, real‐time and fully automated monitoring of multispecies systems. We use case studies from both laboratory‐based and field‐based experiments to demonstrate how data collection can be automated with sensor technologies and robotics, and how collected data can be directly analysed using computer vision and deep learning algorithms. Such frameworks offer the ability to automatically detect, track, count and classify multiple species, but also quantify their interactions, behaviours and morphological traits, at previously impossible resolutions. We then illustrate and discuss how modified versions of these automated frameworks can be operated on various ecological communities to revolutionise their monitoring.

## FROM AUTOMATED DATA COLLECTION TO ECOLOGICAL KNOWLEDGE

### The automation workflow

**FIGURE 1:**
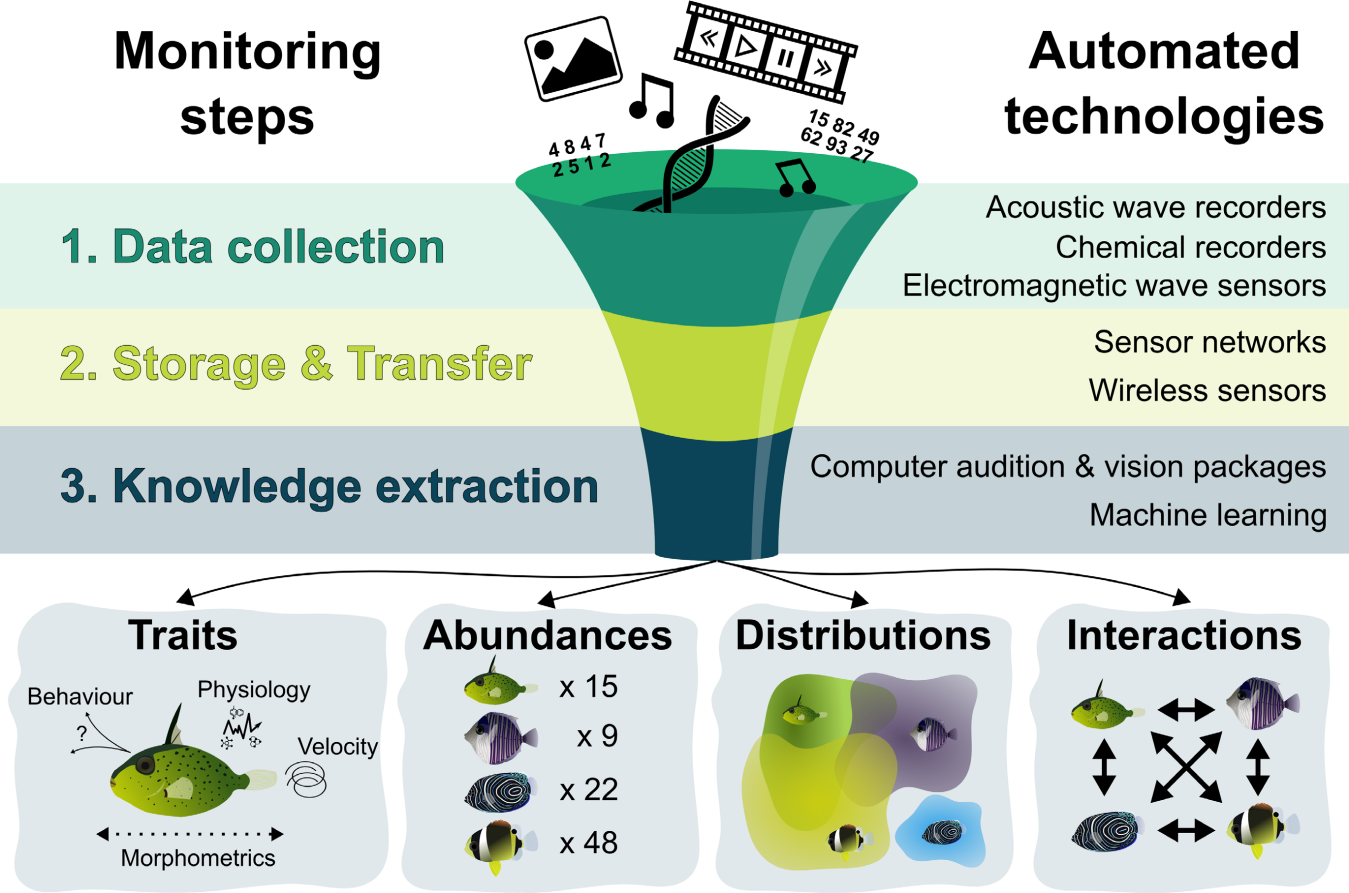
The automation workflow for monitoring populations and communities. From data collection to the extraction of ecological knowledge, a synthesis of the technologies that can automate the acquisition of information regarding individual traits and species abundances, distributions and interactions, which are key metrics for the monitoring of ecological communities.

**FIGURE 2:**
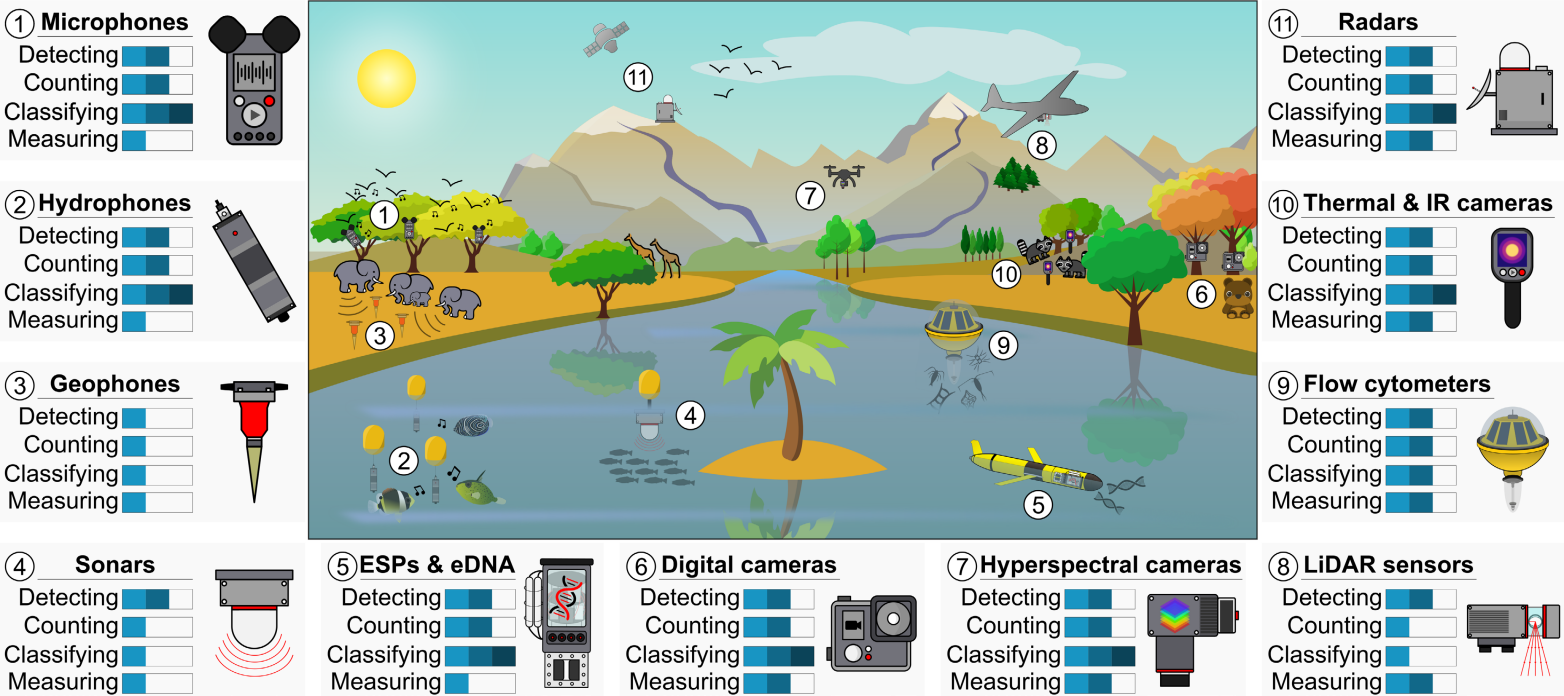
A diversity of automatic recorders to monitor ecological communities non‐invasively and remotely. (1) Vocalising birds being monitored by microphones deployed on trees. (2) Stridulating and drumming fishes being recorded by hydrophones attached to moorings. (3) Walking elephants producing ground vibrations perceived by geophones. (4) Fish shoal being detected by a sonar. (5) Oceanic glider navigating an Environmental Sampling Processor (ESP) to sample eDNA. (6) Bear being detected by camera traps fixed on trees. (7) Hyperspectral camera mounted on a drone and monitoring tree composition in a forest. (8) LiDAR sensor mounted on an unmanned aerial vehicle monitoring multiple forest canopies. (9) Imaging flow cytometer attached to a mooring and recording planktonic communities. (10) Racoons being detected by thermal and IR cameras at night. (11) Stationary radar and a satellite radar, respectively, monitoring bird and large mammal populations. Recorder's ability to detect the presence of living organisms, count their numbers, classify them at the species level and measure their traits (e.g. behavioural, functional and morphological traits) is evaluated from 1 to 3 levels as follows: 1 bar corresponds to ‘in corner‐case situations only’, 2 bars corresponds to ‘in specific conditions and on specific organisms (for detecting, counting and classifying) or for a limited number of features (for measuring)’, and 3 bars corresponds to ‘in most cases and for most organisms (for detecting, counting and classifying) and for several features (for measuring)’.

Automated monitoring of ecological communities requires automating the collection, storage, transfer and processing of data to extract knowledge about the individuals, populations and communities (Figure 1). Among the key metrics that population and community ecologists aim at studying are species presences, abundances, functional traits, distributions and interactions, as well as individual's morphology, behaviour and physiology (Jetz et al., 2019). Myriad automatic recorders can observe the environment non‐invasively and collect data such as sounds and images from which it is then possible to detect, count, classify and measure unmarked organisms (Lahoz‐Monfort & Magrath, 2021). Most of these recorders can be grouped into three main categories: (i) acoustic wave recorders (e.g. microphones, hydrophones, geophones and sonars); (ii) chemical recorders (e.g. environmental sample processors and DNA sequencers); and (iii) electromagnetic wave recorders (e.g. cameras and other optical sensors, LiDAR and radar systems) (Figure 2). Automating the extraction of ecological metrics from such recorders requires storing the data they collect before transferring it to computational platforms that can translate it into ecological knowledge (Figure 1).

### Automatic recorders of ecological information

#### Acoustic wave recorders

**FIGURE 3:**
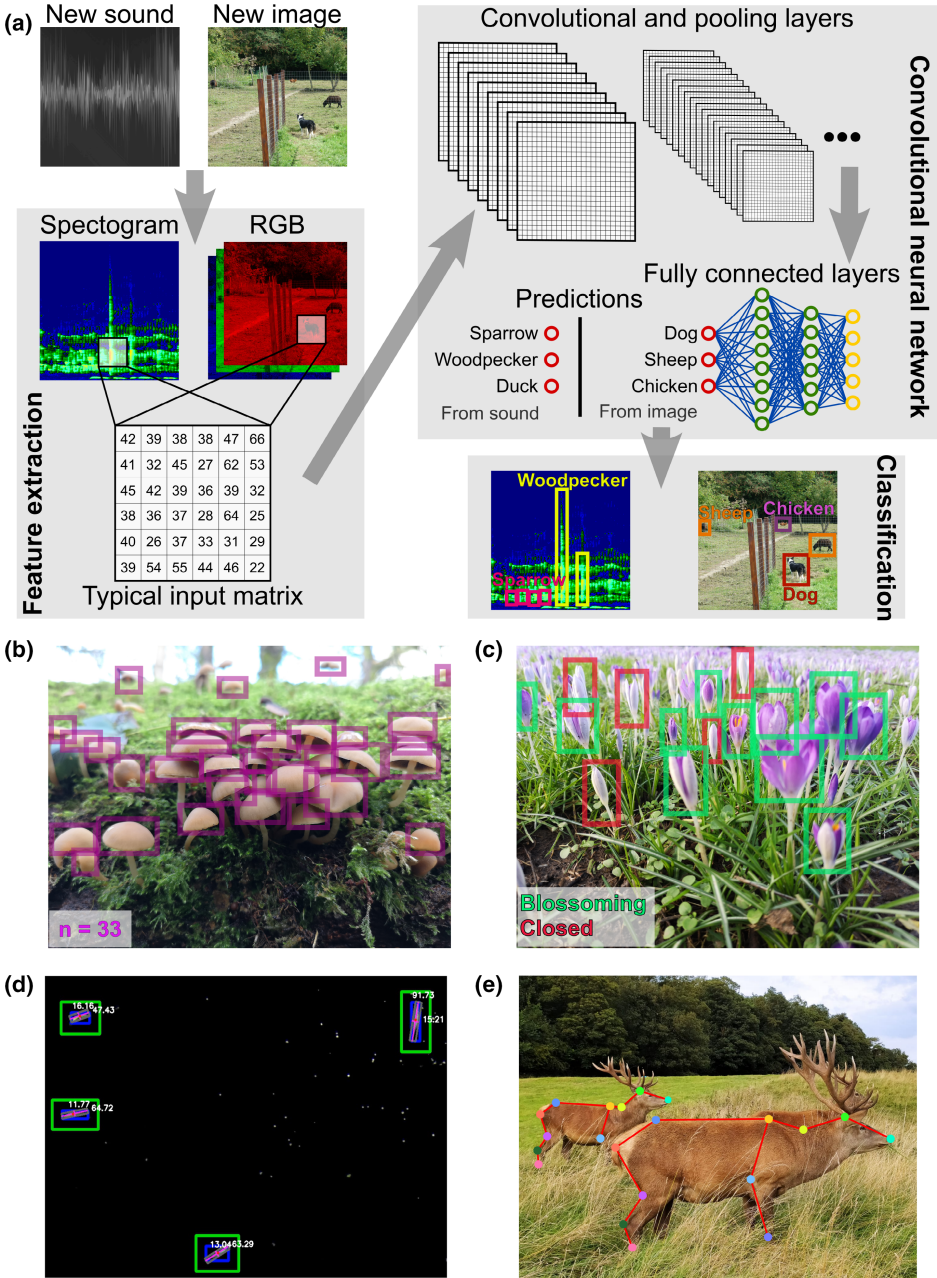
Deep neural networks and their application in monitoring ecological communities. (a) Schematic representation of a convolutional neural network (CNN) architecture and its application to classify multiple species based on sound or image data. (b) Typical example of CNN output when used to count the number of organisms present in an image such as in (Lu et al., 2019). (c) Typical example of CNN output when used to monitor plant status such as in (Mohanty et al., 2016). (d) and (e) represent the output from other types of deep neural networks (i.e. non‐CNN) used to measure organism morphometrical traits such as in (Jung, 2021) and estimate animal pose such as in (Lauer et al., 2022; Mathis et al., 2018; Nath et al., 2019) respectively. Photo credits: Marc Besson.

Microphones, hydrophones and geophones can record the mechanical pressure waves produced by living organisms, such as bird, fish and mammal vocalisations, but also the sounds produced by insect and invertebrate activities, and the ground vibrations generated by large terrestrial mammals (Bradbury & Vehrencamp, 1998) (Figure 2). The audio data obtained by these sensors are called soundscapes, from which one can extract ecological information about the present sound‐producing organisms (i.e. acoustic fingerprints). Computer audition software and machine learning algorithms can be used to analyse the sound frequencies and their amplitudes to detect relevant audio features (Figure 3a), and to translate these features into ecological knowledge (Gibb et al., 2019; Mcloughlin et al., 2019). Indeed, this workflow can be used to automatically flag the presence of a sound‐producing animal (Gervaise et al., 2021; Mac Aodha et al., 2018; Mankin & Benshemesh, 2006), its identity relative to other conspecifics (Favaro et al., 2017), and its behaviour (Ibrahim et al., 2019; Mortimer et al., 2018; Szymański et al., 2021). Acoustic features can also generate estimates of the total number of sound‐producing individuals (Pieretti et al., 2011; Wrege et al., 2017) as well as determine their species identity (Acconcjaioco & Ntalampiras, 2020; Caruso et al., 2020; Kawakita & Ichikawa, 2019; Mukundarajan et al., 2017; Roemer et al., 2021). While being essentially limited to sound‐producing animals (but see Jung et al., 2018 for sound production in plants), passive acoustic recorders could record cryptic species in low‐visibility conditions and over large spatial distances. Moreover, audio data can allow the identification of subpopulations that morphological phenotyping alone cannot discriminate, as evidenced in a damselfish species (Parmentier et al., 2021). However, the quantification of individual morphological traits using audio data is obviously limited. Morphological traits can only be roughly estimated when being directly correlated to an audio feature, for example, when some frequencies or intensities can only be produced by an animal of a certain age/size (Favaro et al., 2017).

In contrast to their passive counterparts, active acoustic recorders first produce sound pulses before listening for the sound echoes being backscattered by the environment and organisms (Benoit‐Bird & Lawson, 2016). Given the rapid and efficient transmission of sounds in water, active acoustic monitoring has almost exclusively been carried out in aquatic environments using sonar technologies (Figure 2). The acoustic features present in sonar echo data can be used to automatically detect small organisms like copepods and krill (Bernard & Steinberg, 2013), and track fish and squids at depths over 800 m (Dunlop et al., 2018; Kay et al., 2022). The taxonomic resolution of sonar remains low, and these technologies are unable to classify most organisms at the species level (Benoit‐Bird & Lawson, 2016). Nevertheless, recent analysis methods based on deep learning have been able to successfully distinguish between echoes from two fish species (Marques et al., 2021) and two krill species (Fontana et al., 2021).

#### Chemical recorders

Living organisms continuously alter the biomolecular composition of their environment, for example through respiration and excretion of faeces, mucus and skin (Taberlet et al., 2018). Among these molecules, DNA contains information specific to species and individuals (i.e. genotypes). Sequencing nucleic acids can inform us about the presence and abundance of organisms in air (Clare et al., 2022; Lynggaard et al., 2022), freshwater (Li et al., 2021), marine (Agersnap et al., 2022; Boussarie et al., 2018) and terrestrial environments (Massey et al., 2022). Environmental DNA (eDNA) metabarcoding has traditionally required large sample volumes and long, labour‐intensive and expensive laboratory operations (e.g. sample filtration, DNA extraction, purification, amplification, sequencing and sequence blasting and alignment) that were not suitable for automated remote monitoring of ecological communities. However, recent developments in miniaturised microfluidic technologies that automate the sampling and processing of eDNA samples (Dhar & Lee, 2018; Formel et al., 2021), and the advent of autonomous vehicles to carry such devices (Yamahara et al., 2019) have enabled the conception of environmental sample processors (ESPs) that can perform all these steps from sampling to DNA amplification and sample storage without human intervention (Hansen et al., 2020; Jacobsen, 2021) (Figure 2). While ESPs do not automate post‐sampling procedures such as DNA sequencing, equipping these devices with modules composed of portable nanopore sequencing devices such as the *MinION* and *SmidgION* (Ames et al., 2021; Jain et al., 2016) could allow them to achieve fully automated status in the future (Huo et al., 2021).

Species chemical fingerprinting is not restricted to nucleic acid and can also be operated on volatile and waterborne organic compounds such as carbohydrates, lipids and peptides. Mass spectrophotometry approaches have successfully been used to identify and classify micro‐organism and plant species using their chemical fingerprint signatures (Emami et al., 2016; Lozano et al., 2022; Musah et al., 2015; Parveen et al., 2020). While being usually cheaper and faster than metabarcoding, these technologies require mass spectra reference libraries, and remain therefore primarily used on laboratory experimental communities (Mortier et al., 2021; Rossel et al., 2019).

#### Electromagnetic wave recorders

Electromagnetic wave sensors capture the electromagnetic energy radiated by the environment, either passively (e.g. digital cameras) or after emitting their own pulses (e.g. LiDAR and radar systems). The most common and affordable of these sensors are digital cameras, which can record images in the visible spectrum. Images produced by digital cameras comprise three matrices of red‐green‐blue (RGB) pixel intensities, from which it is possible to extract image features such as colours, shapes, contours, textures and relationships to surrounding pixels (Weinstein, 2018) (Figure 3a). Using computer vision approaches, including some that involve machine learning (Wäldchen & Mäder, 2018; Weinstein, 2018), these image features can be automatically detected and used to perform individual tracking (Lopez‐Marcano et al., 2021), counting (Lu et al., 2019) and morphological measurements (Kühl & Burghardt, 2013; Mathis et al., 2018; Nath et al., 2019; Pennekamp & Schtickzelle, 2013; Walter & Couzin, 2021), as well as classifying multiple individuals, behaviours and species (Lürig et al., 2021; Weinstein, 2018; Zhou et al., 2021) (Figure 3a–e). Monitoring of larger flora and fauna using ground‐level camera traps is already well‐established (Norouzzadeh et al., 2018; Richardson, 2019; Richardson et al., 2018), and, as resolution and availability of satellite imagery increase, it becomes practical to detect and count megafauna from space (Guirado et al., 2019; Xue et al., 2017). Similarly, the potential for in situ camera‐surveillance of small insects is now apparent, and such approaches may eventually be applicable at microscopic scales (Høye et al., 2020).

When visibility is limited, thermal cameras, active infrared cameras and artificial illumination can help to monitor presence and activity of organisms (McCarthy et al., 2021; Zahoor et al., 2021). By capturing the contrast between the heat (infrared radiation) emitted by organisms and their surroundings to generate an image (Starosielski, 2019), thermal cameras offer the opportunity to detect and quantify the abundance of endotherms in low light conditions (Steen et al., 2012), even when being occluded by vegetation, smoke or fog (Corcoran et al., 2019; Corcoran, Winsen, et al., 2021). However, thermal cameras generally have lower spatial resolutions than standard digital cameras (Christiansen et al., 2014). In contrast to passive thermal cameras, active infrared cameras first pulse short wavelength infrared light before capturing the infrared energy reflected by the environment. Active infrared cameras are not limited to the monitoring of warm‐blooded organisms (Teutsch et al., 2021), and their greater resolution than thermal cameras is useful for species classification (Mu et al., 2019). However, they are more sensitive than thermal cameras to visual noise caused by dust, haze and smoke, which can hide key image features and hamper subsequent image analyses (Soan et al., 2018).

Hyperspectral cameras, often mounted on aerial vehicles, can measure dozens to hundreds of narrow spectral bands from the electromagnetic spectrum. Hyperspectral imagery comprises many matrices (one per spectral band) from which several image features can be extracted (ElMasry & Sun, 2010). This high spectral resolution can be used to predict the chemical composition of subjects observed, allowing, for example, the remote monitoring of plant health status at the individual level (Näsi et al., 2015) and functional traits such as growth, biomass and successional status (Asner, Martin, et al., 2015), with the objective of getting as close as possible to species identification (Dalponte et al., 2012) (Figure 2). The spatial resolution of hyperspectral imagery is usually limited (Feng et al., 2020) but remains well adapted to the monitoring of tree communities from an aircraft flying over large spatial ranges (Miyoshi et al., 2020; Nevalainen et al., 2017; Saarinen et al., 2018).

The lack of spatial resolution from hyperspectral imaging can be compensated by a coupling with digital camera (Feng et al., 2020) and LiDAR (Light Detection and Ranging, or laser imaging) technologies (Cao et al., 2021; Eitel et al., 2016). LiDAR systems actively emit a pulsed laser light and measure its echo using an optical sensor to draw digital 3D representations of the targets based on laser signal return times and wavelengths (Melin et al., 2017). When scanning over a forest, LiDAR provides relatively fine morphological details about tree targets such as height and vertical structure (Vauhkonen et al., 2016). The canopy cover and leaf area index obtained from LiDAR data can be used to infer biomass, growth and assess tree condition (Korhonen et al., 2011; Melin et al., 2017). These morphological features can be combined with the chemical compositions obtained with hyperspectral imagery to build random forest and machine learning‐based classifiers for the monitoring of tree communities at the species level (Cao et al., 2021; Scholl et al., 2021).

At microscopic scales, flow cytometers also use laser imaging, but illuminate particles suspended in a liquid sample with light pulses of various wavelengths to record the phase and intensity of the illuminated particle, from which a hologram and an image can be reconstructed (Işll et al., 2021). Imaging flow cytometers can directly capture images of the passing particles and measure various of their morphological features (Işll et al., 2021). While this technology is primarily limited to the monitoring of microorganisms, it offers the possibility to detect, count, measure and classify high numbers of different particles, and has thus proven to be well suited to the monitoring of planktonic communities such as microalgae and dinoflagellates (Fischer et al., 2020; Pomati et al., 2011). Cytometers can also measure the physiological state of these organisms, such as algal photosynthetic and enzymatic activities (Furuya & Li, 1992; Hyka et al., 2013).

At much larger scales, radars are active electromagnetic ray sensing devices, which scan their environment with micro and radio waves (beyond the infrared in the electromagnetic spectrum), and have been shown to be able to monitor wildlife (Baratchi et al., 2013; Hüppop et al., 2019; Lahoz‐Monfort & Magrath, 2021). For example, by looking at the frequency shift from the transmitted signal to the received signal, pulse Doppler radars can provide information about the biomass, location, velocity and nature of moving birds (Zaugg et al., 2008), fishes (Benoit‐Bird et al., 2003), insects (Hu et al., 2016), and marine mammals (DeProspo et al., 2004). Most radars operate on frequency/wavelength ranges that can penetrate the barriers affecting typical optical sensors. As such, radar technologies are particularly adapted to long‐distance monitoring of flying animals or organisms in the open ocean, but often at low taxonomic resolution (Hüppop et al., 2019) (Figure 2).

### Automated monitoring at scale

The sensing devices introduced so far can either: (i) be deployed locally and individually to monitor a single area; (ii) be assembled in a network to cover larger areas or (iii) be mounted on vehicles to navigate along larger scale transects. By transmitting their respective signals over long distances, single sonars and radars usually cover large areas. These technologies are often fixed, and single units can detect the presence of living organisms thousands of meters away for sonars and up to the continental scale for some radars (Benoit‐Bird & Lawson, 2016; Hüppop et al., 2019). The spatial range of most microphones, hydrophones, geophones and cameras is more limited, from a few centimetres to a few kilometres. Generally, deploying these sensing devices in single and stationary recording stations is sufficient to monitor populations and communities of small body sizes such as ciliates in microcosms (Pennekamp & Schtickzelle, 2013), insect colonies (Tashakkori et al., 2017) or species with clumped distribution patterns such as demersal fishes and small range singing birds (Desjonquères et al., 2020; Frommolt, 2017). When aiming at monitoring communities at a specific place (e.g. wildlife crossing structures and fish aggregating devices), rather than exploring the whole species distribution, single stationary recording units are also appropriate (Brehmer et al., 2019; Fischer et al., 2020; Ford et al., 2009; Pomezanski & Bennett, 2018). In contrast, when aiming at monitoring living organisms with larger home ranges, sensing devices can be assembled in networks (e.g. along transects or within grid‐frameworks). Sensor networks such as microphone arrays can locate vocalising animals by comparing the signal reception timing at different recorders (Sethi et al., 2020; Verreycken et al., 2021). In contrast, cameras have a greater directionality in their sensing, hence deploying numerous cameras in a network can increase the field‐of‐view coverage when being oriented in different directions or placed at different locations (Steenweg et al., 2017). Camera sensor networks can also improve object detection, identification and measurement accuracy when multiple cameras record the same environment from different perspectives (Zhu et al., 2021). Sensor networks can also deploy diverse types of recorders, such as the infrared sensors and digital cameras found in camera traps (Swann et al., 2004), and the multisensory devices used for bat monitoring (Gottwald et al., 2021). While being challenging to implement and automate, units that combine different sensing technologies—for example, combined digital cameras, hyperspectral cameras and acoustic recorders—often provide a more comprehensive and accurate picture of the studied system by capturing more species (e.g. visible and cryptic species) and more data types (e.g. behavioural, morphological, physiological and abundance data) (Chapuis et al., 2021; Frouin‐Mouy et al., 2020; Ireland et al., 2019; Michez et al., 2021; Wägele et al., 2022). When the price of the automatic recorder is prohibitive (e.g. high definition and hyperspectral cameras), deploying multiple units of them in a network may not be feasible. Instead, mounting such recorders on autonomous vehicles such as drones (Corcoran, Denman, & Hamilton, 2021; Gonzalez et al., 2016), oceanic gliders (Kowarski et al., 2020), satellites (Fretwell et al., 2017; LaRue et al., 2017), or terrestrial robots (Bietresato et al., 2016) offers opportunities to automate ecological monitoring over large spatial scales.

A major challenge during automated ecosystem monitoring involves the temporal scales and resolutions over which they must be observed. Long time windows are particularly important when collecting data regarding the dynamic nature of population sizes or seasonal phenology. On the other hand, monitoring individual behaviours and interactions requires high temporal resolution during small time windows. By linking recording devices to power sources such as solar panels and small wind turbines, it is possible to extend their lifetime (Sethi et al., 2018), but these solutions are not applicable in every context, and can increase the operational costs and feasibility of the monitoring program. One way to expand the lifetime of automatic recorders with a limited and finite power supply (e.g. non‐rechargeable battery) is to integrate on‐board processing of data from low‐energy sensors before deciding whether to trigger other power‐hungry sensors. For example, most camera traps are equipped with power‐efficient passive infrared sensors that only trigger high‐resolution video recording when an animal is detected (Welbourne et al., 2015, 2016). The rise of portable electronics has seen the development of affordable and power‐efficient microprocessors (e.g. Raspberry Pi) and microcontrollers (e.g. Arduino). Accessibility of these devices is revolutionising our ability to monitor ecosystems at low‐cost and low‐power consumption (although sleeping in very low power mode cannot be achieved yet for Raspberry Pi) for application in off‐grid locations (Jolles, 2021). These solutions have inspired the conception of low‐cost sensing devices such as AudioMoth (Hill et al., 2018, 2019), HydroMoth (Lamont et al., 2022), Aurita (Beason et al., 2019) and KiloCam (https://www.ecologisconsulting.com/), all of which can operate acoustic and visual sensors, on‐battery and for extended periods of time. For systems installed without any internet access, data need to be collected on external drives, which may need replacing on a regular basis. Using on‐board data processing approaches can minimise storage to only critical and informative components, extending battery life and storage capacity (Liu et al., 2019). For example, time‐lapse wildlife camera systems powered by lithium AA batteries can run remotely for several months without human intervention, except for replacing SD cards (Mann et al., 2022). For systems with internet access, the introduction of 5G cellular networks and specialised networks for the Internet of Things (e.g. Low‐Power Wide‐Area Networks) has facilitated the high‐bandwidth data transfer between recording devices and computational resources (Chettri & Bera, 2020). Such wireless sensors that directly send their recorded data to external servers have the advantage of not being limited by storage capacity and can allow for virtually unlimited continuous monitoring of a system (Sethi et al., 2018). Nevertheless, such frameworks require monitored sites to be equipped with antennas and/or relays, as well as with an energy source to power up data transmission, which are invasive additions to ecosystems (Levitt et al., 2021). Therefore, similarly to abiotic sensor networks, it is important to consider the best practices for network design and sensor data management to minimise impacts on ecosystems and management costs while optimising sensing quality and connectivity (ESIP EnviroSensing Cluster, 2014; Yu et al., 2020).

### Tools for automatic extraction of ecological knowledge

Fully automated monitoring of ecological communities requires computational analysis pipelines that can process and extract knowledge from the large datasets generated by sensing technologies. Diverse computational methods exist for feature extraction, as well as classifying and measuring the characteristics of those features (Lürig et al., 2021). These have recently been dominated and greatly improved by deep learning approaches, based on models such as Convolutional Neural Networks (CNNs) (Brodrick et al., 2019) (Figure 3a–e). Excellent reviews about the usage of deep learning in bioacoustics and computer vision, as well as current trends and limitations already exist (Christin et al., 2019; Gibb et al., 2019; Høye et al., 2021; Mcloughlin et al., 2019; Stowell, 2022; Stowell et al., 2019; Tuia et al., 2022; Wäldchen & Mäder, 2018; Weinstein, 2018). Therefore, reviewing these methodologies and their technical characteristics is beyond the scope of this work. However, there are several key considerations when using machine learning methods in the context of automated analyses in ecology and the types of data captured by remote and distributed sensing systems.

First, effective machine learning models typically require large amounts of training data where a ground truth is known. For imaging datasets, this would involve the annotation of large numbers of images with the specific features that need to be extracted (e.g. classification of individual pixels as ‘organism’ or ‘background’ if segmentation is the goal). While a single training set might allow for a model to accurately extract features for similar types of images, it is rare that a single model can generalise well to vastly different environments. Indeed, generalising animal detection and classification in new locations remain a great challenge, since many state‐of‐the‐art algorithms only perform well on the same location where they were trained (Beery et al., 2018). As powerful as deep learning technologies are, they remain sensitive to distribution shifts between the training data and the data of the downstream use case. Therefore, separate models are often generated for specific use cases with training data sets required for each. In contrast, developing location invariant and robust deep learning classifiers requires infusing data subsets from each location (e.g. images from each camera trap) into the training (David et al., 2020; Shepley, Falzon, Meek, & Kwan, 2021), but can only be achieved by first collecting a larger number of images from numerous and diverse contexts (David, 2021). Doing so for multiple species remains a major challenge that publicly available data sources (e.g. iNaturalist, Pl@ntNet) and easy‐to‐use tools to aid with the often‐manual annotation process can help to address (Lauer et al., 2022; Mathis et al., 2018; Pereira et al., 2019; Shepley, Falzon, Lawson, et al., 2021). However, this step can hamper the application of deep learning approaches in specific areas where existing data is scarce and difficult to gather.

Second, CNNs are well suited to general feature extraction from sensor data where spatial and temporal information is captured by the position of measurements in data matrices. To make sense of this data, CNNs exploit multi‐resolution representations to capture generalised features that can be further combined. In the context of images, this might include at a low‐level being able to distinguish edges by changes in contrast across nearby pixels, while at a high‐level using these edge features to help capture shapes of relevance to specific types of object in a scene (e.g. different organisms). Beyond extraction of simple features like these, processed data can also form input to other analyses such as tracking and interaction mapping algorithms, as well as other machine learning models able to capture higher‐level characteristics (e.g. behavioural traits). Recurrent Neural Networks (RNNs) and Transformer models have become commonplace in natural language processing and image analysis to aid in machine translation (Young et al., 2018) and the understanding of video content (Khan et al., 2022). While their use in ecology to date has been limited, it is likely their application will grow as large multidimensional datasets become available through automated sensing technologies. For example, Transformer models for species classification and distribution prediction from image and sound recordings in the field have already begun to emerge (Conde & Turgutlu, 2021; Elliott et al., 2021; Joly et al., 2021; Reedha et al., 2022).

Third, a feature of deep learning models that is particularly interesting to remote monitoring applications is the efficiency with which data can be processed. Although the training of a deep learning model often requires extensive processing and memory resources, executing a trained model requires only a fraction of this computational power. Furthermore, specialised microprocessors and models are beginning to emerge to efficiently run in low power settings (Lou et al., 2020; Sanchez‐Iborra & Skarmeta, 2020). This has brought machine learning at the place where data collection happens, enabling simultaneous collection and analysis of data and reducing the amount of data that needs to be stored and transmitted (Dutta & Bharali, 2021).

Finally, it is important to recognise that no single machine learning method, nor computer audition/vision package can suit all automated monitoring purposes. Instead, various computational pipelines, each suited to dealing with a specific context or processing step, depending on data types and on the organisms being monitored, are likely to be needed. Contrary to intuition and similarly to sensor deployment and maintenance, the full automation of knowledge extraction from ecological dataset initially depends on people and labour. With labour ranging from data annotation and management to model development, training ecologists for achieving fully automated monitoring of ecological communities might trend towards literacy in the relevant data types, as well as collaborations with engineers and computer scientists. Therefore, we argue that there is a timely need for dedicated funding streams to both train ecologists in these methods and to develop coordinated research networks with such standardised data acquisition protocols. We therefore believe that developing easy‐to‐use systems, with workflows connecting existing machine learning and analysis methods, would help stimulate future research and funding in this domain. This would then allow greater effort to be placed on addressing specific challenges to fully automate ecological monitoring, such as how and when to trigger recordings, deal with data storage and pre‐process data before feeding them into a fully automated analysis program.

## COMBINING TECHNOLOGIES TO FULLY AUTOMATE THE MONITORING OF MULTISPECIES SYSTEMS

### Fully automated monitoring of micro‐organisms in experimental systems

Experimental laboratory systems have been used for decades to examine how individual morphological traits, species abundances, distributions and interactions respond to various stressors. Collecting such data is often time consuming and labour‐intensive, which limit data resolution and replication. Nevertheless, experimental systems ensure controlled environment (e.g. lighting conditions that guarantee species visibility), calibration of—and unlimited power‐supply to—high‐definition automatic recorders such as modern digital cameras. Therefore, laboratory systems are often the initial developmental space for automated technologies, and help pioneering technological advancements that can later be transferred into the field (Joska et al., 2021). Small‐scale experimental systems offer a perfect opportunity to test and develop the concept of fully automated workflows (Alisch et al., 2018). Here, we detail a system developed to collect multidimensional data on freshwater protists, ciliates and rotifers to evaluate the resilience of these ecological communities in response to biotic and abiotic stressors (Box 1).

BOX 1 **Automated pipeline for monitoring freshwater protists in experimental microcosms**

**FIGURE 4:**
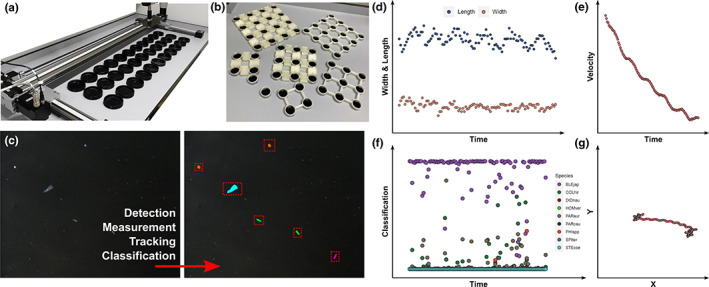
Overview of a fully automated workflow towards the monitoring of multidimensional data from multispecies protist communities in experimental systems. (a) Robotic gantry navigating a microscope and camera over experimental microcosms. (b) Examples of other microcosm landscapes that can be used within this workflow. (c) Video analysis workflow, from raw frames to measurement and classification of moving objects using the CNN classifier. Red bounding boxes indicate the detected individuals and coloured overlay indicate different species. (d–g) Length and width, velocity, classification and trajectory measurements, respectively, obtained by this automated workflow for a single moving object (i.e. protist organism) over the duration of the video. Photo credits: Marc Besson.

1. *System presentation*
  The system is composed of a robotic gantry that controls the *X* and *Y* positions of a 6K‐14fps camera mounted on a stereomicroscope, navigating over 3D‐printed experimental landscapes (i.e. microcosms, Figure 4a,b). This set up allows videos of each microcosm to be automatically collected and analysed to extract information about the abundance and distribution of species, and individuals' morphological and behavioural traits (e.g. size, velocity, turning rates) (Besson et al., 2021a, 2021b).
2. *Automated video acquisition*
  The robotic gantry and the camera are controlled by an in‐house‐developed Python program that consists of the following steps (i.e. first part of the automation workflow):

- Parametrisation: A two‐column data frame containing the *X* and *Y* locations where we want to move the gantry is loaded into the program (i.e. gantry location loop). A one‐column data frame containing the GMT times for which the gantry will loop over the different locations is loaded into the program. Video duration is then selected, as well as a file path for where to save the video that will be recorded by the camera.
- Video acquisition loop: The gantry then starts its loop at the times indicated in a. The gantry moves the camera to the first *X*/*Y* location and starts the camera. A first check controlling whether the camera is well positioned over a microcosm is performed using another in‐house‐developed OpenCV algorithm (Bradski, 2000). If the position is not correct, the gantry moves the platform until the camera field of view matches with a microcosm. Once the position is correct, a second check is performed by reading a QRcode fixed to the microcosm. This QRcode contains information about the microcosm ID, treatment and replicate, which are stored as variables to properly name the video that is then recorded. Once the video is saved, the camera turns off and the gantry moves to the next location, repeating this same procedure.
- End of the loop: Once all locations have been navigated to, the gantry moves back to its home location, before starting the video acquisition loop again as many times as listed in the time data frame loaded during the parametrisation step.

3 *Automated video analysis*
  The second part of the automation workflow consists of processing and extracting ecological knowledge from the videos that were automatically acquired. To achieve this, we firstly developed a computer vision methodology using OpenCV in Python (Bradski, 2000). Since the model species are in constant motion, we used background subtraction to segment objects corresponding to living organisms from the background (Figure 4c). The segmented objects are then measured (e.g. centroid location, length, width, surface area, orientation) using basic OpenCV functions, and tracked using a custom algorithm based on Kalman‐Filtering (Patel & Thakore, 2013). Tracking objects allows us to calculate morphometric, velocity and trajectory metrics for each segmented object over the entire video (Figure 4d–g). Classifying objects by assigning them a species name is operated by sending each object's images into a CNN based on MobilenetV2 and pretrained on ImageNet (Deng et al., 2009; Howard et al., 2017). This CNN was fine‐tuned using automatically generated training protist image datasets that we obtained by recording videos of single species and using the same segmentation/tracking methodology described above. Classifying all objects in all frames allows the collection of multiple classification data: one per frame for each single object, increasing classification accuracy by looking at the classification time series of each object (Figure 4f).

This workflow combines robotic, camera and deep learning technologies to fully automate the monitoring of protist communities over days to weeks, allowing the collection of multidimensional data (i.e. behavioural and morphological traits for each individual, and abundances and distributions for each species) at resolutions that would be impossible to achieve manually (one data per frame, at more than 10 frames per second). This pipeline allows to play with multi‐patch landscapes (Figure 4b), thus exploring the effects of landscape fragmentation, patch connectivity and multiple stressors induced at the patch level, on protist community dynamics (Clements et al., 2014; Clements & Ozgul, 2016). Ongoing upgrades of this system will equip every patch with miniaturised abiotic sensors such as temperature, oxygen and pH probes, to have a more comprehensive monitoring of each of these microscopic ecosystems. This workflow is generally well‐suited to the monitoring of microorganisms (e.g. freshwater and marine phyto‐ and zooplankton), and can be easily adapted to larger experimental systems such as mesocosms, macrocosms and in the field (e.g. over water tanks, river streams, green houses, aviaries and fields). This would allow to scale at the community level the existing automated single species monitoring, such as those existing for ants and fish in the laboratory (Cao et al., 2020; Lopez‐Marcano et al., 2021), and directly in the field (Francisco et al., 2020; Imirzian et al., 2019), by adding a species classification layer to these automated frameworks. Furthermore, such larger‐scale systems would allow the use of multiple cameras without the need to mount them on a microscope and robot, reducing the cost of the recording part of the monitoring system while allowing to record of multiple landscapes simultaneously.

### Fully automated monitoring of plant–pollinator interactions in field mesocosms

Reports of drastic declines in insect diversity, abundance and biomass carry severe implications for ecosystem services such as pollination. However, insects are difficult to study, and traditional methods require substantial manual effort to collect data. Data are particularly sparse and patchy in logistically challenging areas. As a result, there are large geographic and taxonomic gaps in data about insects—including many pollinator—preventing thorough investigation of the drivers and severity of insect declines. Cameras and computer vision can help to solve data deficiencies in entomology and pollination, enabling remote data collection and automatic identification at unprecedented spatial and temporal resolutions (Høye et al., 2021). For some research questions related to plant–pollinator interactions, such as characterising plant phenology, recording must be hourly or even daily, while for questions related to pollination events or pollinator–pollinator interactions, the framerate must be in seconds or even milliseconds. Here, we detail a system to enable fully automated in situ monitoring of plants and pollinators at these resolutions, including inter‐ and intra‐specific interactions (Box 2).

BOX 2 **Automated pipeline for monitoring plant–pollinator interactions in field mesocosms**

**FIGURE 5:**
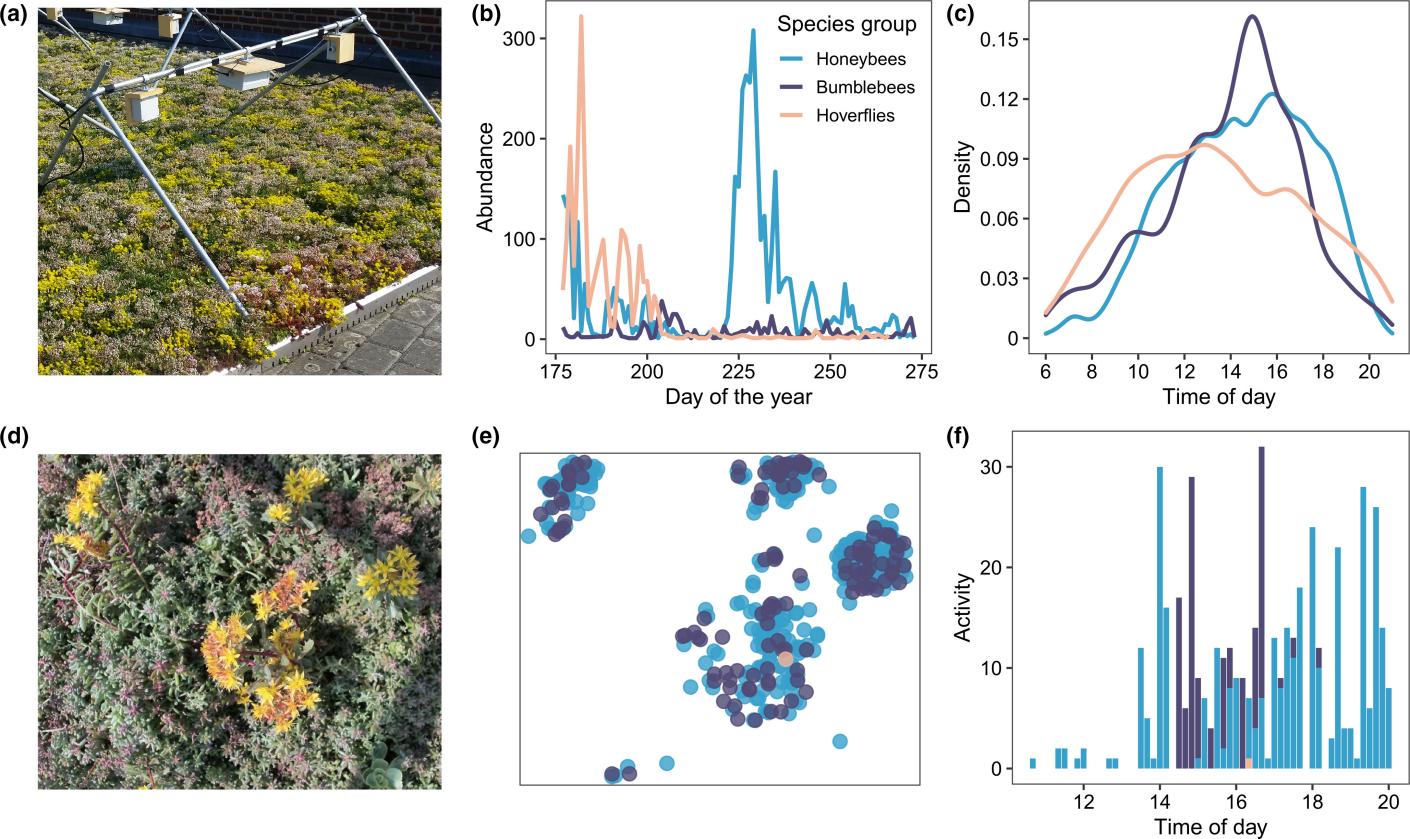
Insights from real‐time, fully automated in situ monitoring of plants and pollinator interactions. (a) The automated pollinator monitoring system records a green roof comprising *Sedum* flowers. (b) Continuous surveillance allows the annual phenology of different pollinator groups to be quantified at fine temporal resolutions (blue = honeybees; dark purple = bumblebees; light orange = hoverflies; abundance = number of individual tracks). (c) Diurnal phenology can also be compared across groups, showing a relative preference of hoverflies for mornings and honeybees for evenings. (d) Image from day 234 of 2020, a day of high pollinator activity. (e) Activity of different insect groups on day 234 can be mapped to inflorescences in (d) to quantify plant–pollinator interactions. (f) Real‐time monitoring even allows exploration of pollinator‐pollinator interactions; the activity (total detections) of honeybees, bumblebees and hoverflies is shown for 10‐min intervals during day 234, where bumblebees are only active during a remarkably short period of the day.

1. *System presentation*
  The system collects non‐invasive, high‐resolution data on flowers and pollinating insects. High‐volume acquisition of images to train CNNs is achieved using a camera, mounted on a steel frame, with a power supply and a memory storage unit. By incorporating computers into on‐site hardware, CNNs can then detect, classify and track insects and flowers in real‐time. The system allows rigorous monitoring of abundance, diversity and phenology of plants and pollinators. By automatically generating entomological data at unprecedented spatial and temporal resolutions, real‐time tracking can revolutionise our understanding of not only plant–pollinator interactions, but pollinator–pollinator interactions.
2. *Automated image acquisition*
  Affordable webcams (e.g. Logitech C920 HD Pro Webcam ~$60) and wildlife time‐lapse cameras (e.g. Wingscapes TimelapseCam Pro ~$150) have been successfully deployed in this system. Key image acquisition parameters include frame rate, recording periods, focal distance and resolution. These parameters are adjusted based on the study system and the mode of data collection. High‐volume data collection generates the imagery needed to train a CNN, while real‐time data collection leverages those CNNs to collect entomological data at extremely high frequency.
  - High‐volume data collection aims to generate a representative image library for off‐site annotation and analysis. It is appropriate for pollination systems which lack trained CNNs, if data storage and labour are not strong limiting factors. Frame rate and recording periods are limited by storage capacity on‐ and off‐site, as well how frequently storage and power can be replenished. Recording 12 frames every hour to a 128 gb SD card at 4224 × 2376 pixel resolution, including LED flash at night, 20,000 images can easily be recorded over 70 days (Wingscapes TimelapseCam Pro with one set of eight AA lithium batteries).
  - Real‐time data collection, defined here as processing in parallel with image capture, builds upon resources generated by high‐volume data collection. It involves rapid on‐site analysis of images, retaining only text‐based detection data and a subset of images for validation. Having massively reduced demand for memory, real‐time data collection is very useful for remote sites, provided that trained CNNs are available for detection and classification. It also records flowers and insects at extremely high temporal resolution, allowing in‐depth analysis of individual behaviours and species interactions.
3. *Automated image analysis*
  Image analysis comprises two stages—detection and classification, followed by individual track identification. For detection and classification of flowers and pollinators, image‐series spanning full growing seasons are processed by CNNs. Bjerge et al. (2021) demonstrate automated detection and classification of insects in an urban ecosystem with eight classes of arthropods, including species important for pollination. Using the CNN darknet53 (YOLOv3), they achieve real‐time detection and classification at 0.33 frames per second. Tracking the movement of individuals within the frame can be achieved based on minimal displacement and size‐change of objects between frames (Bjerge et al., 2021). As with automated monitoring of protists (Box 1), a tracking algorithm permits recording of behavioural metrics, but also improved classification. For example, a majority vote can be taken across consecutive classifications of an individual insect. A tracking algorithm can also be deployed to identify and separate individual flowers; this allows derivation of flower‐level data on floral traits, phenology and visitation (Mann et al., 2022).
  Such real‐time detection and classification present exciting opportunities to examine species interactions at unprecedented spatiotemporal resolutions. First, this approach can inform as to whether different pollinator taxa are active during different seasonal or diurnal periods, while accounting for multiple counting of the same individual by counting tracks rather than detections (Figure 5b,c). Similarly, this approach can provide a high spatial resolution view of how different floral resources are used by different insects (Figure 5d,e). Moreover, this approach reveals highly complex short‐term patterns of co‐occurrence, in which different pollinators that are generally active at similar times of day potentially exclude one another on timescales from minutes to seconds (Figure 5f). Such opportunities to quantify fine‐scale pollinator–pollinator interactions are particularly relevant given mounting concerns about the impacts of managed honeybees on wild pollinators (Ropars et al., 2022; Thomson, 2016). Collection of sufficient real‐time data will even allow individual tracks to be examined in relation to the presence and absence of other individuals or species. In this way, we may begin to grasp the behavioural mechanisms behind competitive exclusion as never before.

Using a similar pipeline, plant phenology is being recorded by cameras in environments such as the Arctic as well as along elevational gradients. For instance, cameras record arctic flowers such as *Dryas integrifolia* and *Silene acaulis* to characterise plant and pollinator phenology in extreme environments (Mann et al., 2022). Across Scandinavia, this system helps to understand effects of landscape composition and competition on pollination. In the UK, the system is being extended to monitor pest control in crop fields, showing clear potential to monitor ecological processes such as herbivory, predation and detritivory at hitherto impossible sites and resolutions. Ultimately, automated vehicles, such as drones, could enable in situ camera‐based monitoring of a huge variety of smaller plant, animal and fungal communities. Still, the full potential of automated in situ pollinator monitoring has not yet been realised. Importantly, real‐time data collection is contingent on reliable CNNs, which must be trained on huge numbers of annotated images from relevant ecological contexts. Automated monitoring is thus limited by (1) uptake of standardised, high‐volume data collection with time‐lapse cameras, (2) standardised annotation of massive image libraries and (3) robustness of detection and classification algorithms to novel insects and novel backgrounds. Annotation of insects and flowers can be increasingly outsourced to citizen science platforms such as eButterfly (https://www.e‐butterfly.org/), the Global Biodiversity Information Diversity (https://www.gbif.org/), iNaturalist (https://www.inaturalist.org/), Pl@ntNet (https://plantnet.org/) and Zooniverse (https://www.zooniverse.org/ ). However, a major challenge is the generalisation of such automated solutions to a wide diversity of natural ecosystems. Specifically, even with an exceptionally large training dataset, the system will encounter unfamiliar species, including some that are inseparable within high‐resolution imagery. Three emerging approaches will help this challenge to be overcome: (1) open‐set classification can allow specimens, even those that are not present in training data, to be classified to the lowest possible taxonomic level (Lee et al., 2018); (2) synthetic image datasets can be generated using images of specimens with validated species‐level identification (Skovsen et al., 2020) and (3) combination of data from multiple sources or sensors for species‐level labelling and classification—for example, images may be complemented by DNA sequence data (Badirli et al., 2021).

### Towards the fully automated monitoring of any community in any ecosystem

**FIGURE 6:**
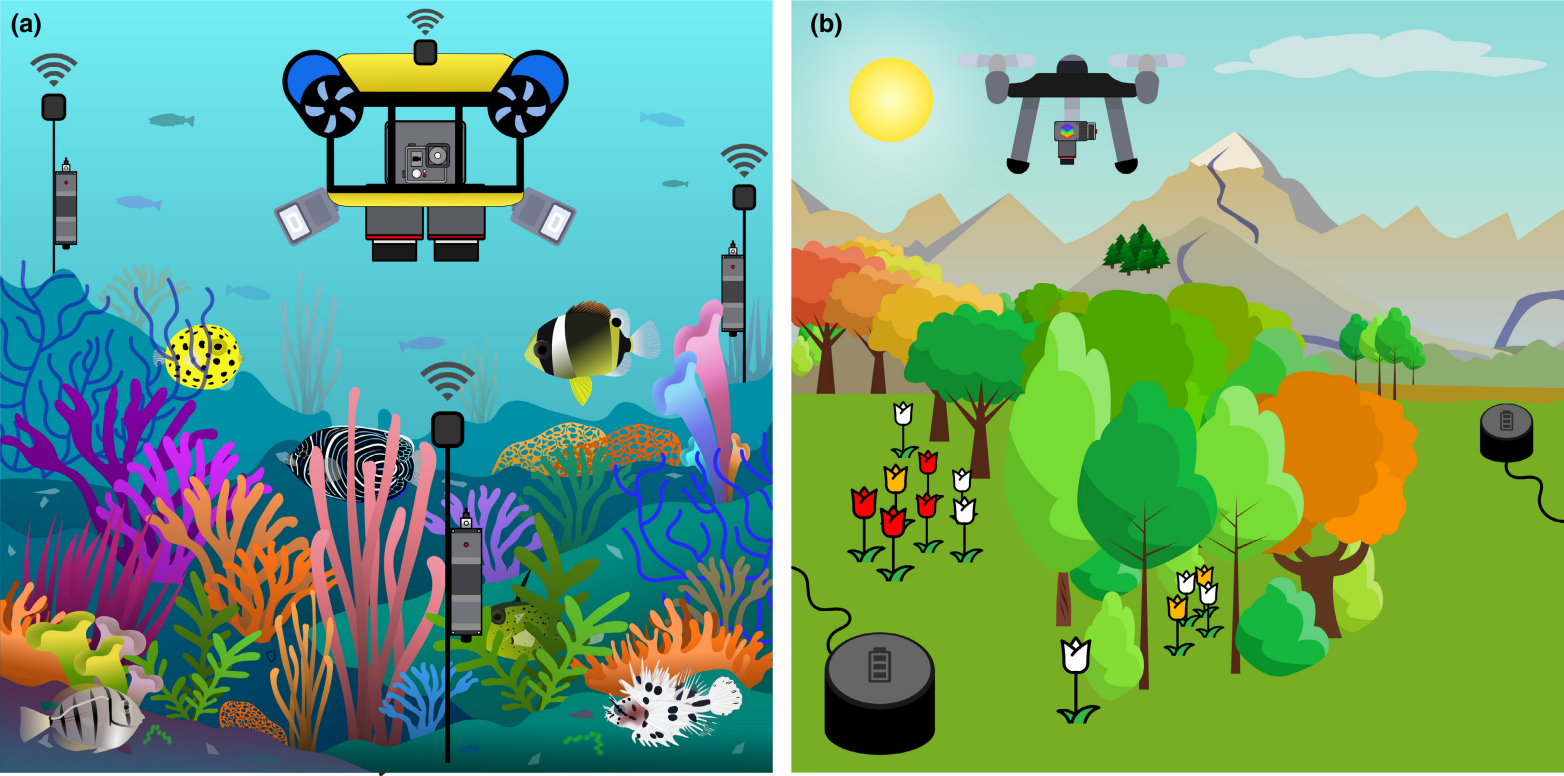
Futurist examples of fully automated wildlife monitoring programs. (a) Autonomous and wireless underwater vehicle equipped with multiple high‐resolution cameras and hydrophone array, together monitoring multidimensional data about coral reef communities such as habitat complexity, coral species distribution and fish functional diversity. (b) Autonomous and self‐charging drones equipped with LiDAR and hyperspectral cameras for the monitoring of plant and tree flowering phenology.

The previous examples of fully automated monitoring of multidimensional data from multispecies systems pose the question of whether such frameworks could be developed for almost any other ecosystem. A first limitation is obviously the requirement for large and properly labelled training datasets when implementing accurate and reliable deep learning classifiers, preventing the monitoring of communities for which such data is not available (McKibben & Frey, 2021). Second, the environmental complexity of natural systems could hamper such designs, generating data with a very low signal‐to‐noise ratio in comparison with experimental systems. Nevertheless, initiatives aiming at capturing and monitoring wildlife habitat complexity do exist and represent great research avenues to combine automated wildlife monitoring and habitat mapping within complex environments. For example, the 100 Island Challenge (https://100islandchallenge.org/) associates classical field surveys with innovative imaging and data technologies to reconstruct 100 m^2^ coral reefs digitally and in 3D, from which all corals are individually annotated and classified at the species level (Naughton et al., 2015). By combining this workflow and the vast amount of labelled 3D coral structures it has generated with approaches aiming at automatically classifying 3D objects such as MeshCNN (Hanocka et al., 2019) and Global point Signature Plus & Deep Wide Residual Network (Hoang et al., 2021), we could automate coral habitat mapping in the future. Moreover, the development of autonomous underwater vehicles would help achieving automated surveys (Modasshir & Rekleitis, 2020; Ordoñez Avila et al., 2021), while coupling these surveys with acoustic monitoring would scale up our understanding about how coral habitats promote surrounding biodiversity (Lin et al., 2021) (Figure 6a).

Another field of research where a combination of novel sensing and machine learning approaches is beginning to bear fruit is forest ecology. With growing access to high‐resolution remote sensing imagery, we have witnessed rapid improvements in algorithms developed to reliably and accurately identify individual trees in both LiDAR point‐clouds and RGB orthophotos (Brandt et al., 2020; Dalponte & Coomes, 2016; Ferraz et al., 2016; Weinstein et al., 2020). Particularly promising are recent efforts to use deep learning to delineate individual tree crowns in RGB imagery acquired from unmanned aerial vehicles (UAVs) and satellites, and then apply them at broad spatial scales (Brandt et al., 2020; Weinstein et al., 2020). For example, *DeepForest* was recently used to map the crowns of around 100 million trees across the National Ecological Observatory Network (Weinstein et al., 2021), while Brandt et al. (2020) used a similar approach applied to sub‐meter resolution satellite imagery to identify around 1.8 billion individual trees spread across 1.3 million km^2^ in the West African Sahara and Sahel. Delineating the crowns of individual trees not only allows us to count their numbers, but also measure key axes of their size that directly scale with their biomass—such as height and crown area (Jucker et al., 2017; Marconi et al., 2021). Moreover, by fusing individual tree maps with multi or hyperspectral imagery, one can also classify individuals to species and estimate several key traits related to plant growth and function (Asner, Anderson, et al., 2015; Asner, Martin, et al., 2015; Dalponte & Coomes, 2016). Generating these baseline distribution maps is the first step towards developing automated routines for tracking forest phenology and dynamics at seasonal, inter‐annual and even decadal time scale. For instance, daily 3‐m resolution PlanetScope satellite imagery trained against data from in situ PhenoCam networks can detect the timing of key phenological stages in the canopy, such as bud burst, flowering and leaf drop (Dixon et al., 2021; Moon et al., 2021). Using RGB cameras mounted on UAVs, with repeated data acquisition over several years also allowed to track forest dynamics (e.g. canopy gap formation), phenology (e.g. leaves and flowering time) and the underlying mechanisms behind treefall rates that traditional survey could not capture (Araujo et al., 2021; Park et al., 2019). Similarly, the use of wireless sensor networks comprising multispectral cameras, LiDAR and abiotic sensors connected to solar‐powered batteries allows to track in real‐time changes in tree growth and key physiological parameters such as water use and local microclimate that are transforming our understanding of processes that constrain when and how fast trees grow (Etzold et al., 2022; Valentini et al., 2019; Zweifel et al., 2021). This information is critical for being able to predict how trees might respond to extreme climate events and for parameterising more realistic global vegetation models (Zuidema et al., 2018). Therefore, when coupled with data from camera traps and acoustic networks (Deere et al., 2020; Sethi et al., 2020) these novel data streams to study plant phenology and forest ecology would allow to (i) build a detailed picture of the interactions occurring in complex vegetated ecosystems across multiple trophic levels; (ii) elucidate how they shift from season to season and year to year and (iii) predict how they will change under novel climate (Figure 6b).

### Towards new ecological knowledge and conservation challenges

Overall, the different monitoring technologies presented here show clear advantages over traditional survey methods, including precise traits estimation, less disturbance (but see below), the ability to cover greater, more remote and potentially dangerous areas, in a repeatable, quantifiable, high‐resolution and standardised way to measure myriad of biological and ecological metrics. Such systems, through their standardisation and the high‐resolution multidimensional data they can acquire, have the potential to generate novel ecological insights (Tuia et al., 2022; van Klink et al., 2022). For example, 24‐hour camera surveillance of Swiss alpine meadows recently revealed moth pollination of *Trifolium pratense*, a phenomenon overlooked during a century of research into that important wildflower and forage crop species (Alison et al., 2022). Furthermore, automation allows ecological interactions to be rigorously quantified at unprecedented spatiotemporal resolutions—ranging from ephemeral interactions between micro‐organisms or insects, to drawn‐out conversations between humpback whales (Cholewiak et al., 2018). Understanding interactions between species and individuals is crucial to predict ecosystem responses to anthropogenic drivers.

The high‐resolution and multidimensional data which can be generated using automated frameworks (e.g. behavioural and morphological traits, abundances and distributions across multiple species) offer the opportunity to develop new predictive frameworks, which for the first time can synthetise data across ecological scales (from individuals to populations) and help developing novel early warning signals that precede population and community collapses (Cerini et al., 2022). Indeed, ecological forecasting is an area where automated frameworks offer significant opportunity, as the resolution of data required to develop robust predictive tools is most often impossible to obtain with non‐automated methods (Cordier et al., 2018; Darras et al., 2019; Lamprey et al., 2020; Marcot et al., 2019; Wearn & Glover‐Kapfer, 2019; Welbourne et al., 2015). Moreover, automated methods allow the acquisition of these data in real‐time, pushing ecological research from the post hoc era to one where forecasts about ecosystems fate are continually updated based on the current observed state, similar to weather forecasting (Deyle et al., 2016; Huang et al., 2019; Slingsby et al., 2020). The step change offered by such real‐time data, in combination with cutting edge statistical methods such as Bayesian statistics and machine learning tools, which both leverage past state to improve predictive accuracy, offers perhaps the greatest opportunity for ecology to become a truly predictive science.

These outstanding perspectives brought by the fully automated, high resolution and multidimensional monitoring of ecological communities should not eclipse the potential negative effects of these technologies on wildlife. For example, unmanned and self‐navigating devices such as drones can affect animal physiology (Ditmer et al., 2015) and behaviour (Bennitt et al., 2019; Bevan et al., 2018; Mulero‐Pázmány et al., 2017; Schroeder et al., 2020), although these disturbances may be less detrimental than those caused by traditional survey methods, with less impact per unit of data (Aubert et al., 2022; Christiansen et al., 2016; Gallego & Sarasola, 2021) and some species becoming rapidly habituated to the presence of unmanned vehicles (Ditmer et al., 2019). Nevertheless, it is timely to (i) better quantify these impacts to avoid the generation of biased and unstandardised data; and (ii) aim to minimise these impacts to prevent animal stress. Ways to mitigate these impacts include the development of new unmanned aircraft systems, such as miniaturised drones and blimp‐like aerostats, which eliminate noise disturbance to wildlife (Adams et al., 2020; Kuhlmann et al., 2022). When such devices are not available, disturbances can be avoided by using greater camera resolutions and obtaining the necessary permits to increase flying height (Scobie & Hugenholtz, 2016). For the autonomous monitoring of the canopy health state and plant phenology, most terrestrial robots are equipped with large wheels or wheel‐chains (Bietresato et al., 2016), which can damage the vegetation (Stager et al., 2019). Legged robots would minimise these impacts, but their development for ecological monitoring is still in its infancy (Gonzalez‐De‐Santos et al., 2020). Similarly, underwater autonomous vehicles have potential to damage underwater vegetation, which, in turn, can clog and strangle propellers (Pedroso de Lima et al., 2020). In addition to the vehicles carrying them, sensor technologies themselves can negatively affect wildlife, as evidenced with sonar technologies on marine mammals (Harris et al., 2018; Southall et al., 2016), and artificial light on insects (Jonason et al., 2014; Kalinkat et al., 2021). Sensors themselves may also be perceived by subject organisms. For example, cameras based on their appearance, sound, flash and even active infrared emissions, may be recognised and consequently alter animal behaviours (Caravaggi et al., 2020). At end‐of‐life, and when being damaged by weather conditions and animas themselves, systems also have the potential to pollute the environment (e.g. via batteries) (Rysgaard et al., 2022). In this context, the development of biodegradable sensing systems represents a promising research avenue (Sethi et al., 2022). Thus, whilst the impacts of automated approaches are often localised, minimal and almost certainly sub‐lethal, they will of course scale with the extent of the sensory network. As such, we propose a cautious rollout of automated monitoring over the coming decades, with concurrent studies aiming to minimise the disturbance caused by automated monitoring apparatus.

## CONCLUSION

Technologies such as automatic recorders and deep learning have not reached their full potential to support modern ecological monitoring in a fully automated manner (Hampton et al., 2013; Tuia et al., 2022). In practice, automation of particular steps of a workflow and subsequent scaling up most often still require substantial labour input, for example, for maintenance and data retrieval. The development of fully automated frameworks may also be limited by challenges associated with building interdisciplinary collaborations among ecologists, electronic engineers and artificial intelligence specialists, to train the specialised staff needed to develop and maintain accessible automated systems (Pedroso de Lima et al., 2020). By synthesising the variety of existing automated technologies and describing real‐world and futurist workflows that bring them together, we aim to stimulate such collaborations in the future—towards the development of new, user friendly and standardised pipelines that automatically monitor multiple components of multispecies systems with minimal disturbance exerted (Weinstein, 2018). The fully automated monitoring frameworks that we present here integrate novel hardware and software approaches allowing the rapid generation of high resolution, multidimensional data across complex ecological communities. In the current era of global change, such data will be critical to (i) reliably compare ecological communities globally and monitor their temporal dynamics, (ii) feed mechanistic models to better predict their fate, (iii) investigate potential signals preceding the changes in the functioning and structure of a system and (iv) examine which stressors are impacting wildlife the most, and which populations are the most at risk.

## AUTHOR CONTRIBUTIONS

Marc Besson and Christopher F. Clements conceived the idea. Marc Besson wrote the paper with significant contributions from all authors. Marc Besson and Jamie Alison designed the figures.

### PEER REVIEW

The peer review history for this article is available at https://publons.com/publon/10.1111/ele.14123.

## Data Availability

Data sharing is not applicable to this article as no new data were created or analysed in this study.
